# Development and Validation of an LC-MS/MS Method for the Quantitative Determination of Contezolid in Human Plasma and Cerebrospinal Fluid

**DOI:** 10.3390/ph16010032

**Published:** 2022-12-26

**Authors:** Guanxuanzi Zhang, Na Zhang, Liuhan Dong, Nan Bai, Yun Cai

**Affiliations:** 1 Medical School of Chinese PLA, Graduate School of Chinese PLA General Hospital, Beijing 100853, China; 2 Center of Medicine Clinical Research, Department of Pharmacy, Medical Supplies Center, PLA General Hospital, Beijing 100853, China

**Keywords:** contezolid, CSF, LC-MS/MS, plasma

## Abstract

To develop and verify a liquid chromatography-tandem mass spectrometry (LC-MS/MS) method for determining contezolid in plasma and cerebrospinal fluid (CSF). Protein precipitation was performed on samples using linezolid as the internal standard. We used an Agilent EclipsePlus C18 column operating at 0.4 mL/min in conjunction with acetonitrile and water mobile phases for the LC-MS/MS analysis. Using the precursor-product ion pairs 409.15→269.14 (contezolid) and 338.14→195.1 (linezolid), multiple reaction monitoring was used to quantify the compounds. Plasma linearity range was 50.0 to 5000 ng/mL, and CSF was 20.0 to 1000 ng/mL (*r*^2^ = 0.999). The inter-batch and intra-batch precisions were ≤2.57% and ≤5.79%, respectively. Plasma recovered 92.94%, and CSF recovered 97.83%. Plasma, CSF, hemolytic plasma, and hyperlipidemic plasma all showed a coefficient of variation ≤ 7.44%. The stability and dilution integrity of this method were also acceptable. The study also demonstrated that artificial CSF can be used as a matrix for the preparation of standard curve samples. A simple and accurate method was developed and validated for the determination of contezolid concentrations in human plasma and CSF, which may be useful for monitoring the therapeutic effect of central nervous system medications.

## 1. Introduction

Antimicrobial resistance in bacteria is becoming increasingly serious, especially in methicillin-resistant *Staphylococcus aureus* (MRSA), methicillin-resistant coagulase-negative *Staphylococci*, penicillin-resistant *Streptococcus pneumoniae* (PRSP), vancomycin-resistant *enterococci* (VRE), vancomycin-intermediate *Staphylococcus aureus*, and vancomycin-resistant *Staphylococcus aureus* [1,2]. The treatment and control of infectious diseases are faced with severe challenges [3].

Oxazolidinone is a kind of synthetic antimicrobial agent [4]. These drugs have a unique mode of action, targeting a unique region of 23S rRNA adjacent to the peptidyl transferase center of the 50s ribosomal subunit, inhibiting protein biosynthesis in bacteria and used to treat severe infections caused by Gram-positive pathogens [5,6,7,8]. Linezolid is the first member of the oxazolidinone antibiotics and has good antibacterial activity against many important Gram-positive bacteria [9,10]. However, potential neurotoxicity and hematotoxicity, as well as significant inhibition of monoamine oxidase, limited the wide use of linezolid [11]. The homology between the human cell mitochondrial protein synthesis pathway and the bacterial ribosomal target also inhibits human cell mitochondrial protein synthesis, which could lead to lactic acidosis, myelosuppression, anemia, thrombocytopenia, pancytopenia, and other serious adverse drug reactions [12]. Contezolid (S)-5-([isoxazol-3-ylamino]methyl)-3-(2,3,5-trifluoro-4-[4-oxo-3,4-dihydropyridin-1(2H)-yl]phenyl), a new oxazolidinone antimicrobial agent, is an innovative antibiotic developed in China and has independent intellectual property rights. Its main active structure is the same as that of linezolid. Studies in vitro showed that contezolid had good antibacterial activity against MRSA, PRSP, and VRE and had no cross-resistance with existing antimicrobial agents [13,14]. Compared with linezolid, it shows better safety as well as minimal myelosuppression and inhibition of monoamine oxidase, which are independent adverse reactions associated with linezolid therapy [13]. Therefore, contezolid may provide a promising alternative therapy for infections with multidrug-resistant Gram-positive bacteria [15].

The methodology for analyzing contezolid in human body fluids has not been extensively studied, and only in two articles is it described how ultra-performance liquid chromatography-mass spectrometry can be used to analyze contezolid concentrations in human plasma and urine [16,17]. The method for the analysis of contezolid in human cerebrospinal fluid (CSF) has not been studied. Infections of the central nervous system (CNS) can be extremely harmful. These lead to fatal outcomes and long-term neurological problems in survivors, including cognitive deficits and motor impairments [18]. Pathogens with Gram-positive characteristics are common etiologic agents of infections of the CNS, including brain abscesses [19]. Antibiotics should be present at therapeutic concentrations at the site of infection if they are to be effective against CNS infections. But the existence of the blood-brain barrier made it difficult for antibiotics to enter the CNS, and it was difficult to obtain effective antibacterial concentration in the CSF and brain tissue, which seriously affected the antibacterial effect and resulted in poor treatment results, even led to ineffective treatment results [20]. Therefore, for new antimicrobials, it is necessary to develop a method for quantitative determination of CSF concentration, which is helpful to explore their efficacy in the CNS.

Our previous study has successfully verified the feasibility of liquid chromatography-tandem mass spectrometry (LC-MS/MS) for the analysis of drug concentrations in human plasma and CSF [21]. The objective of this study is to develop a method for the analysis of contezolid in human plasma and CSF by LC-MS/MS and to verify the methodology to provide an analysis method for the study and application of the drug in treating CNS infections.

## 2. Results and Discussion

This section may be divided into subheadings. It should provide a concise and precise description of the experimental results, their interpretation, and the experimental conclusions that can be drawn.

### 2.1. Method Development

At present, there are no known LC-MS/MS methods for the determination of contezolid in CSF. In this study, we propose to develop a method for the quantitative determination of contezolid in CSF by LC-MS/MS, except for plasma.

#### 2.1.1. Chromatography and MS Conditions

Several C18 columns and several gradient programs were tested for the chromatographic separation of target drugs. The Agilent Eclipse Plus C18 column (100 × 2.1 mm, 3.5 μm) provided the best chromatographic performance for all compounds with appropriate peak shape and sharpness. Moreover, the addition of 0.1% formic acid (FA) to the mobile phase acetonitrile: water (90:10, *v*/*v*) and the compatibility of the chromatographic column enabled the target compounds to be effectively retained and separated by chromatography. The Agilent Eclipse Plus C18 column showed durability and robustness under these conditions.

For MS conditions, usually when the polarity of the analyte is high, electrospray ionization (ESI) is selected. Generally, acidic compounds, which are compounds containing -OH, -COOH, and phenolic compounds, are determined by the negative ESI mode, and basic compounds, which are compounds containing more heteroatoms and nucleosides, are determined by the positive ESI mode. Contezolid has high polarity and is a basic compound containing N, so the ESI and positive ion modes were selected.

In order to optimize chromatographic separation, a series of preliminary experiments were carried out to test different mobile phases, including ammonium acetate, acetonitrile, the mixture of acetonitrile and ammonium, or water, as well as different concentrations of mobile phase additives, such as FA and acetic acid. In the positive ESI mode, the addition of FA reduced the tailing of the peak and improved the response of the target compounds. Finally, 0.1% FA in 10 mM aqueous ammonium acetate (A) and 0.1% FA in 5 mM ammonium acetate acetonitrile: water (90:10, *v*/*v*) solution (B) were selected as the best mobile phase.

For the purpose of establishing the multiple reaction monitoring (MRM) scanning mode, the product ions of each analyte were characterized by product ion scanning under 30 eV collision energy. The product ion spectra of each analyte were obtained. For all analytes, the two most abundant product ions were used as quantifier ions and qualifier ions, respectively. Quantitative determination was carried out by the *m/z* transitions 409.15→269.14 for contezolid and 338.14→195.1 for linezolid.

#### 2.1.2. Sample Preparation

The preparation of samples through effective extraction steps is a common step for the determination of compounds in biological matrices. Yet the cost of the nitrogen used is high, and liquid-liquid extraction is relatively time consuming and difficult to operate. In this work, we considered the method of protein precipitation for sample preparation. Using methanol and acetonitrile as protein precipitates, the matrix effect and extraction recovery were investigated. The results showed that the method had satisfactory recovery and almost no matrix effect and could be used for the determination of clinical biological samples. Of course, this method also had some shortcomings. For example, it was difficult to remove salts and lipids from the matrix, which was easy to interfere with and affect the reproducibility and accuracy of the results. Moreover, the non-specific precipitation reaction might cause the loss of trace analytes along with the co-precipitation of matrix proteins. But the method had a simple operation procedure, shortened the extraction time, and saved the cost. It was suitable for hospitals to analyze clinical biological samples with large size.

### 2.2. Method Validation

#### 2.2.1. Calibration Curve

The calibration standard curves of three different batches were prepared for linear evaluation. Two set of calibration curve samples were prepared freshly on the day of analysis. Each calibration curve consisted of a double blank sample (without analyte or internal standard, IS), and a single blank sample (only IS). Eight concentration levels (50.0, 100, 250, 500, 1000, 2500, 4000 and 5000 ng/mL) were used; the linearity range of plasma was between 50.0 and 5000 ng/mL. Eight concentration levels (20.0, 40.0, 100, 200, 400, 600, 800 and 1000 ng/mL) were used; the linearity range of CSF was between 20.0 and 1000 ng/mL. The calibration curve was generated by the response of peak area ratio (y) and analyte concentration (x), and the linear 1/*X*^2^ weighted relation was used for regression. We also evaluated the batch size, and the results showed that each batch of plasma could be injected 136 times and CSF could be injected 101 times.

#### 2.2.2. Specificity and Selectivity

**Figure 1:**
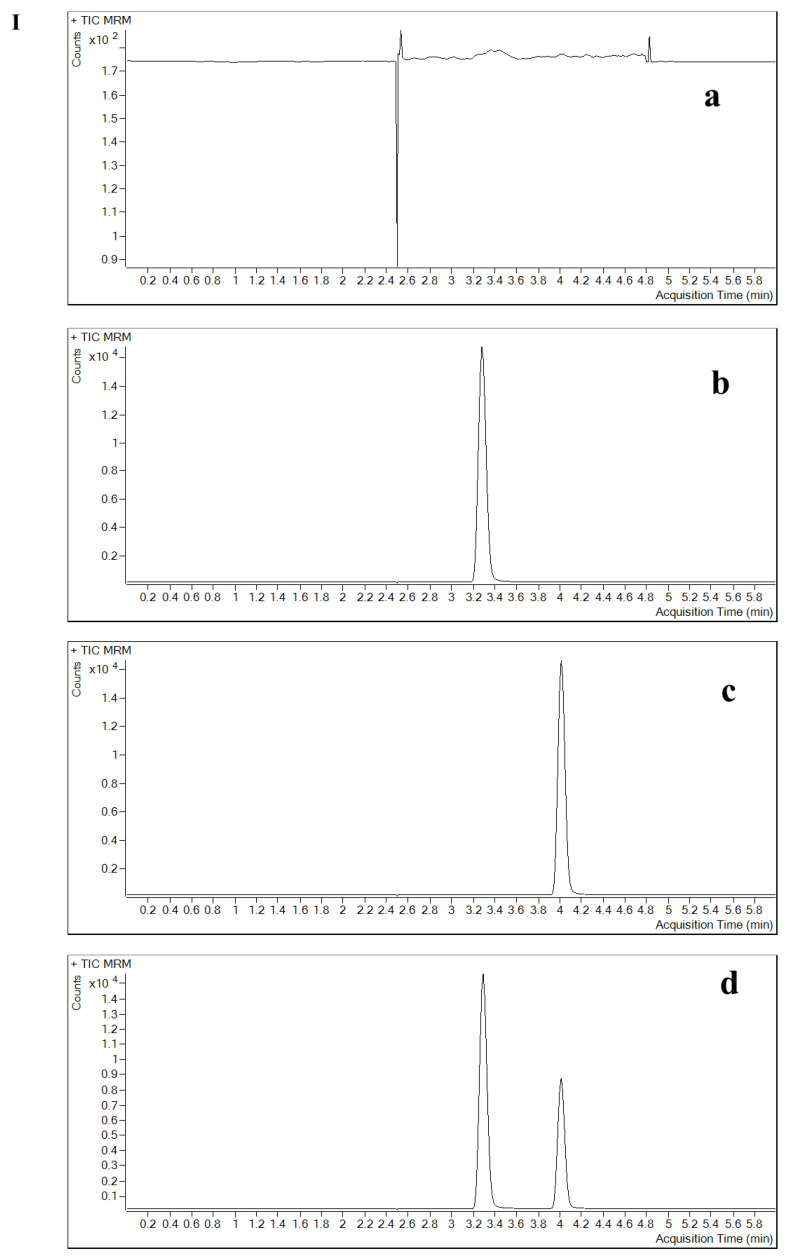

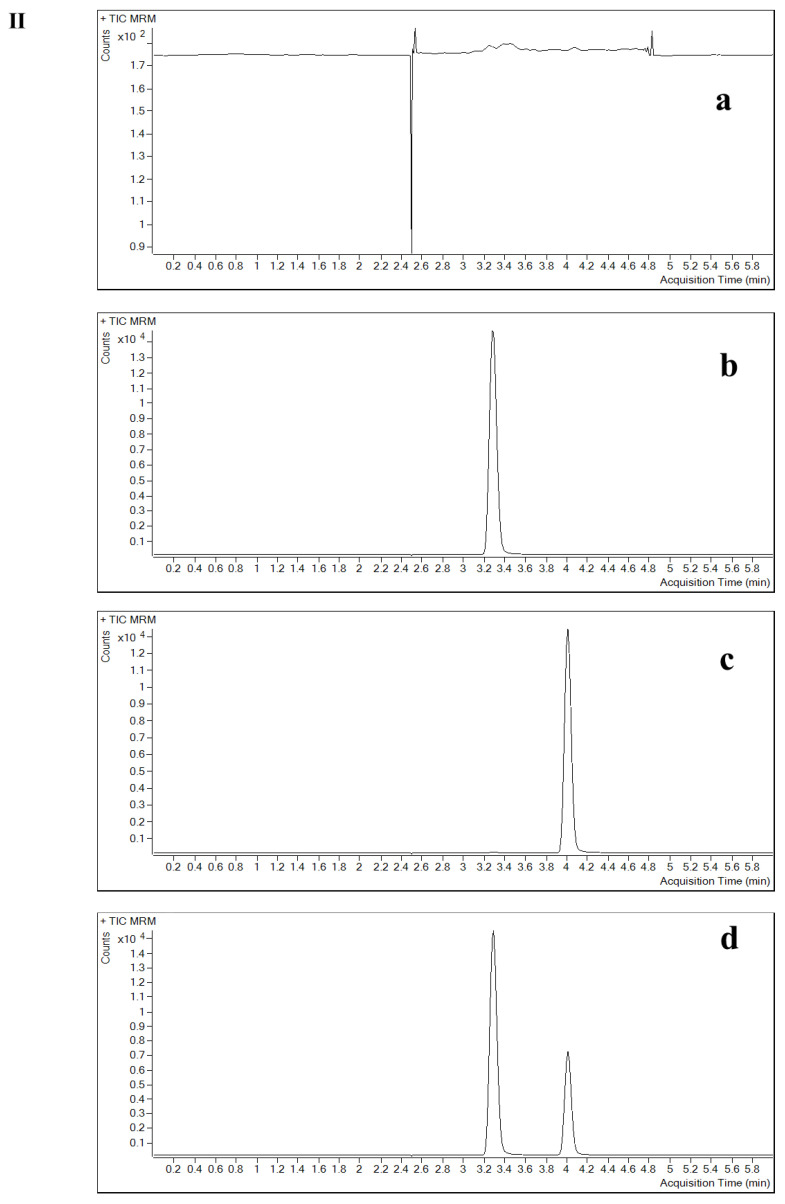

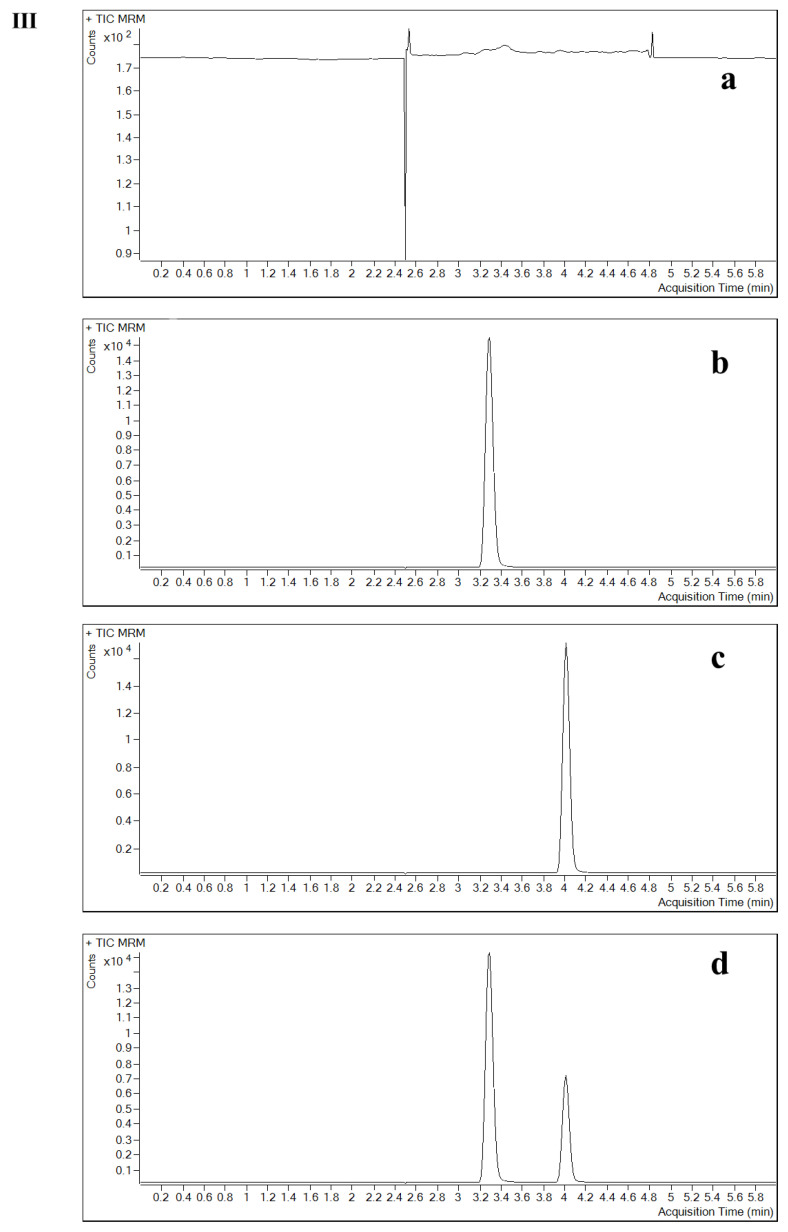

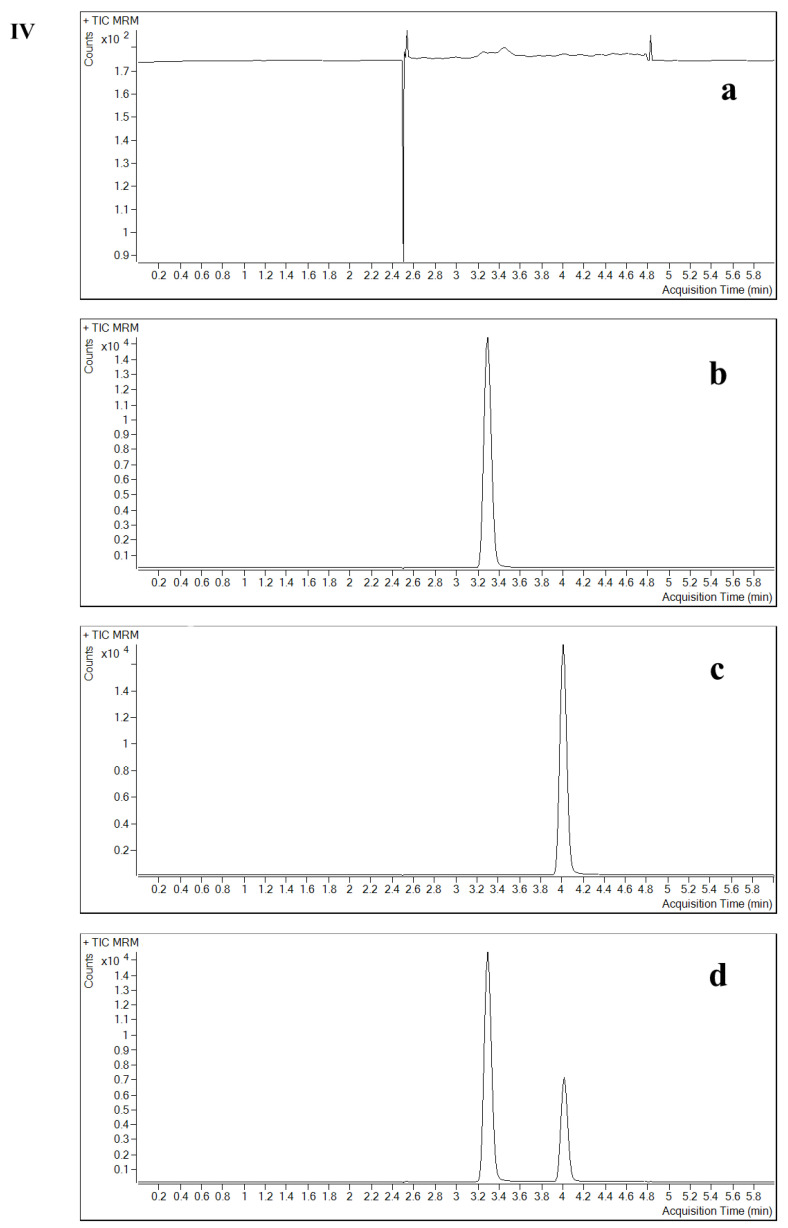

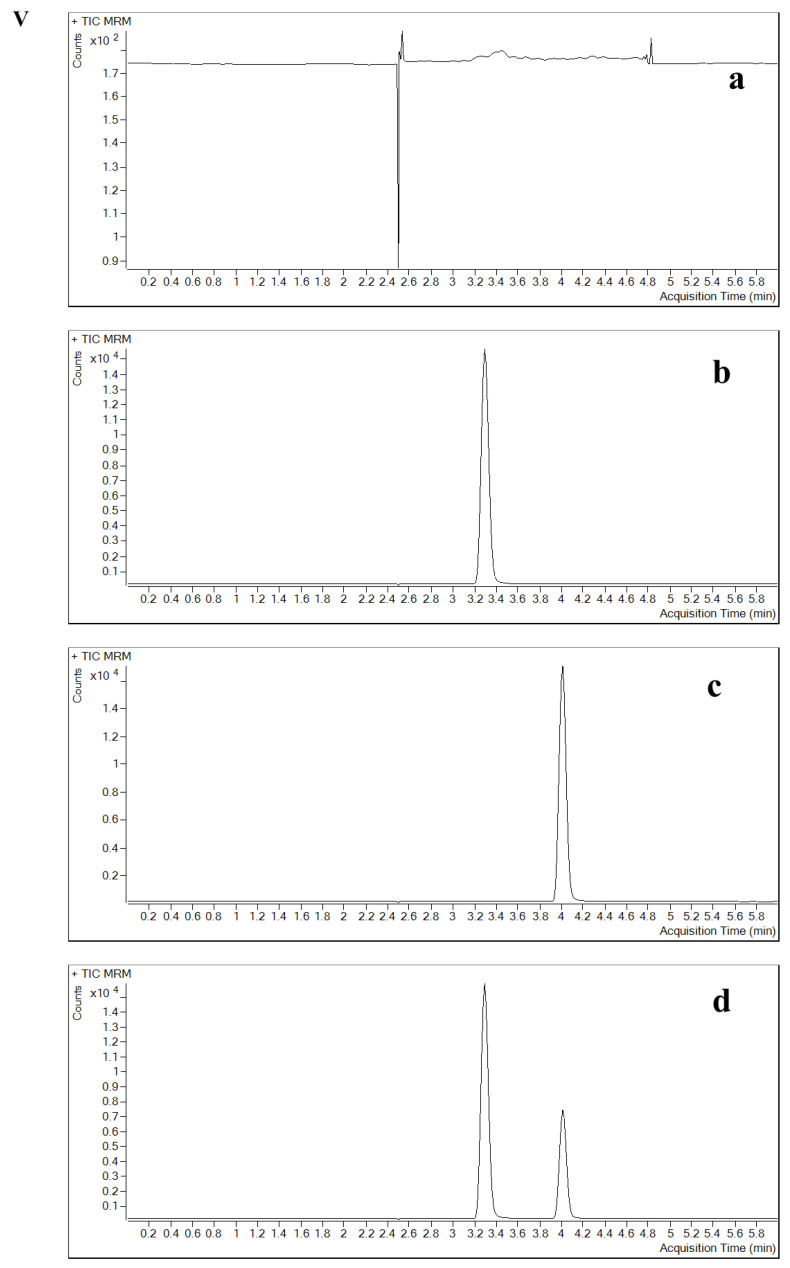

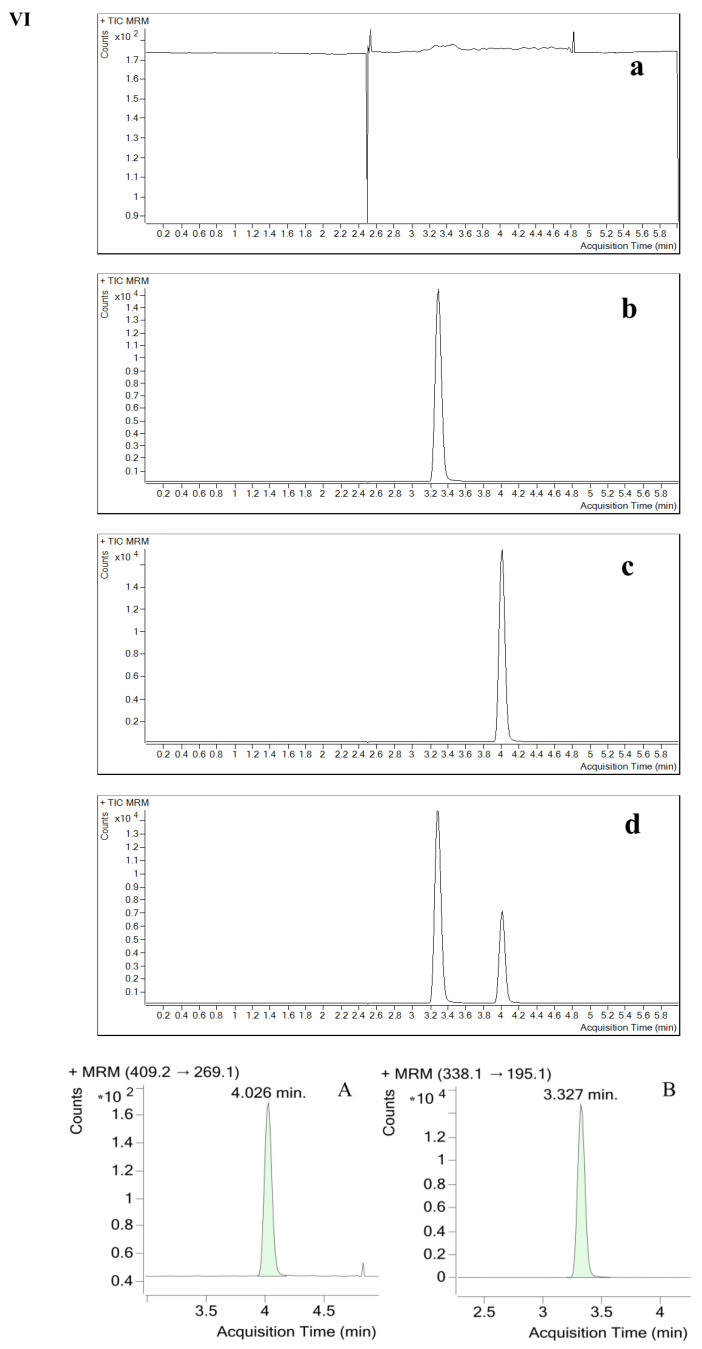
Specificity and selectivity of the method in plasma. (**a**) double blank, (**b**) single blank, (**c**) analyte only and (**d**) analyte and IS (in six different lots). Chromatograms of contezolid (**A**) and linezolid (**B**).

**Figure 2:**
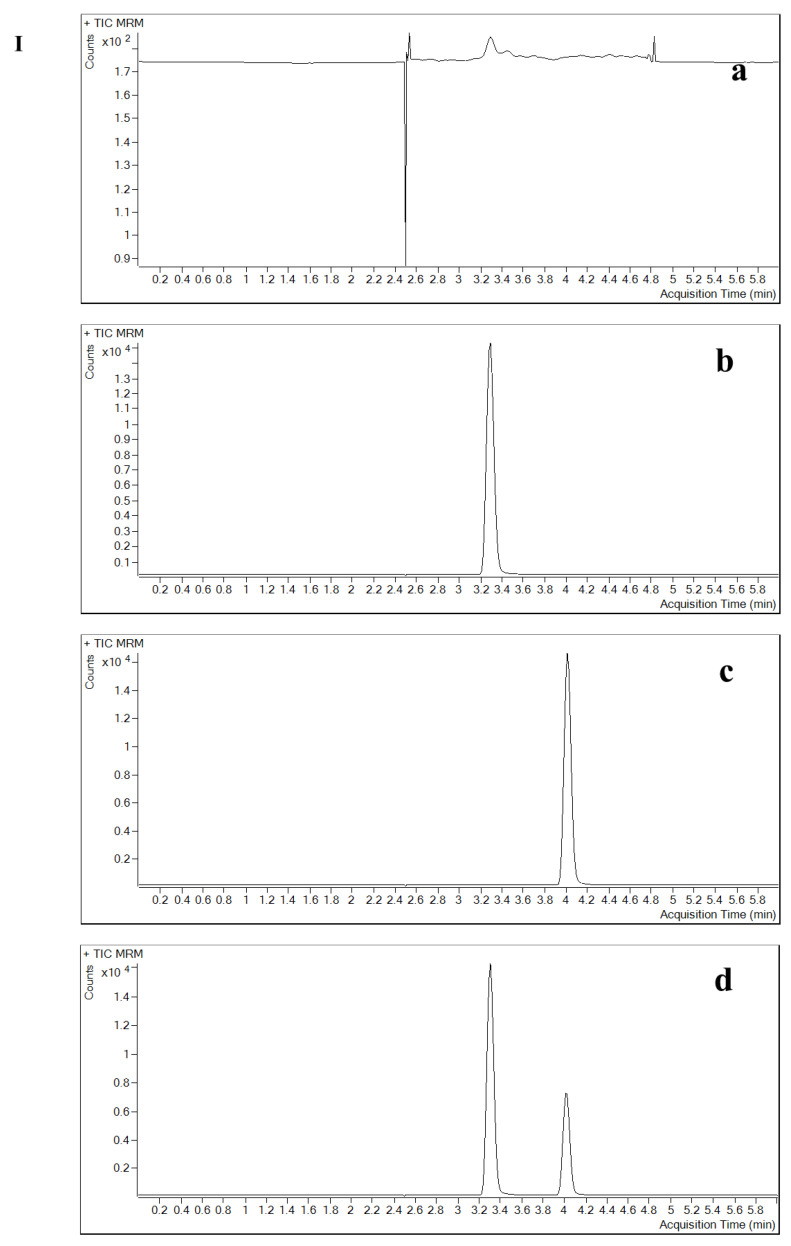

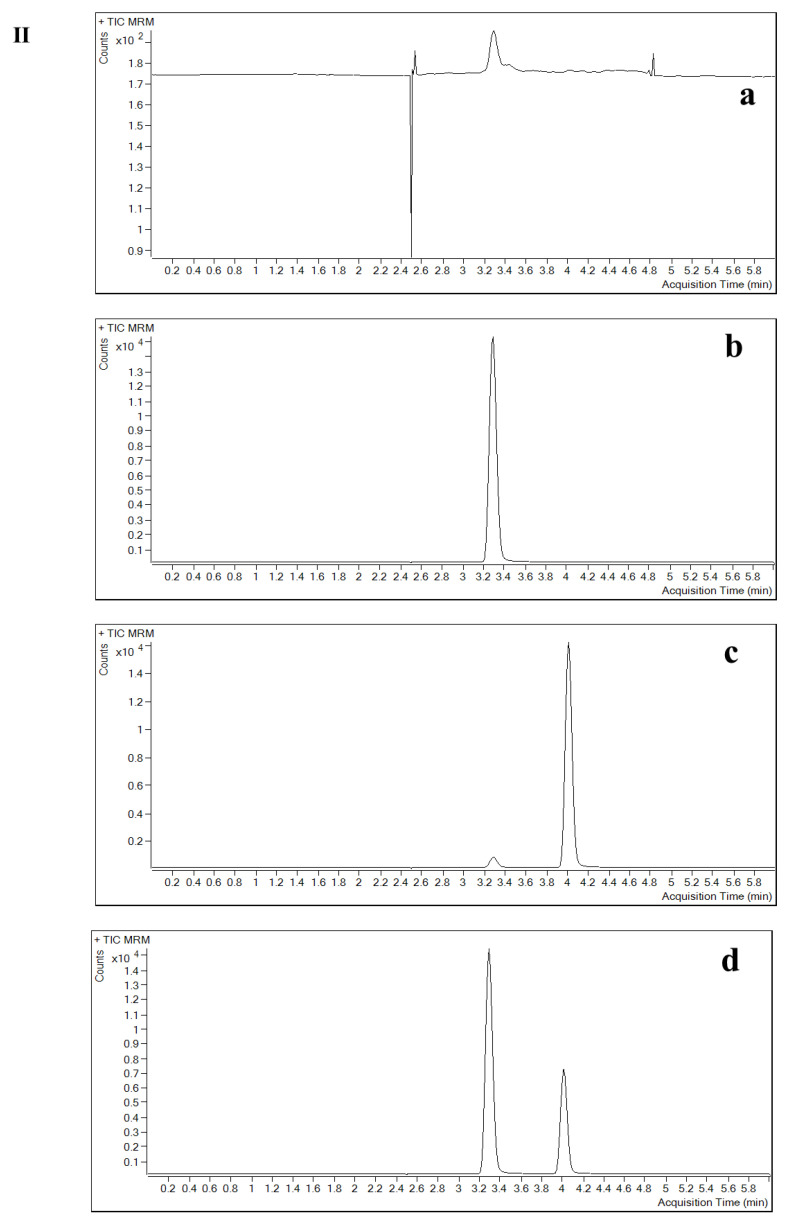

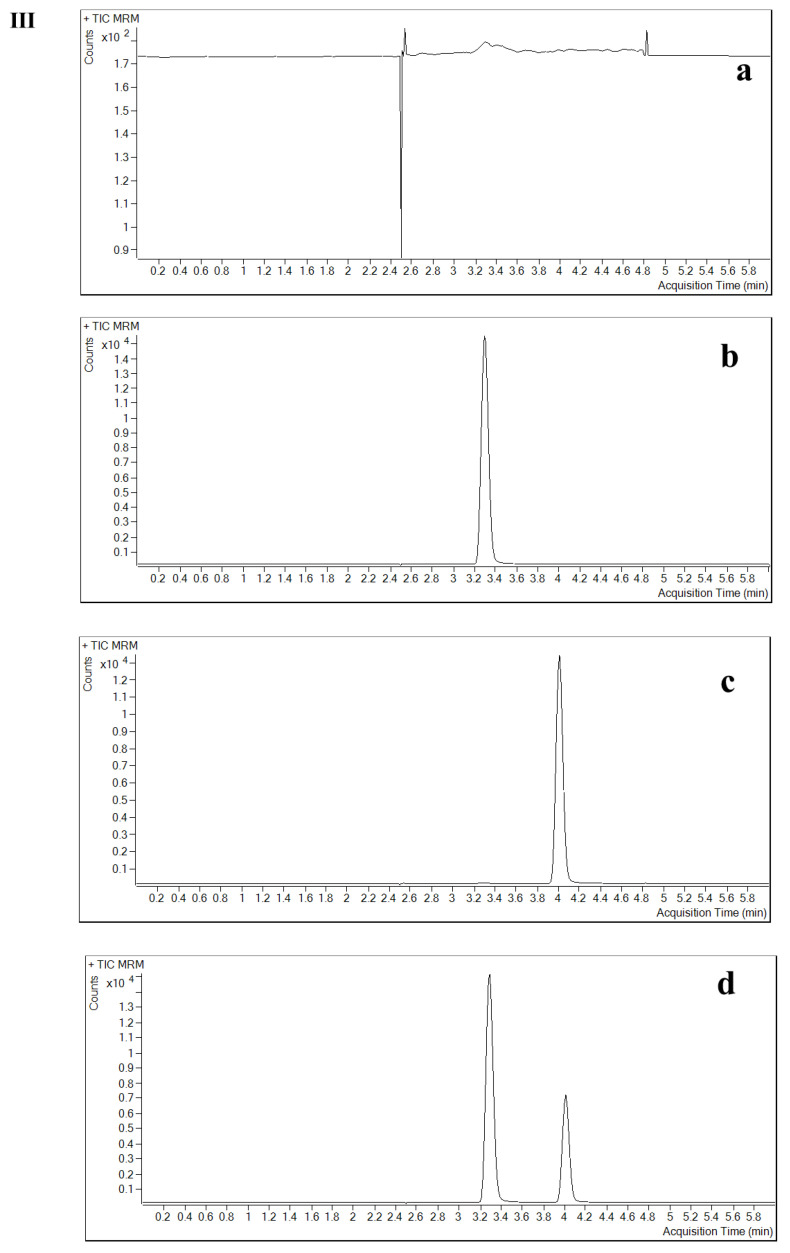

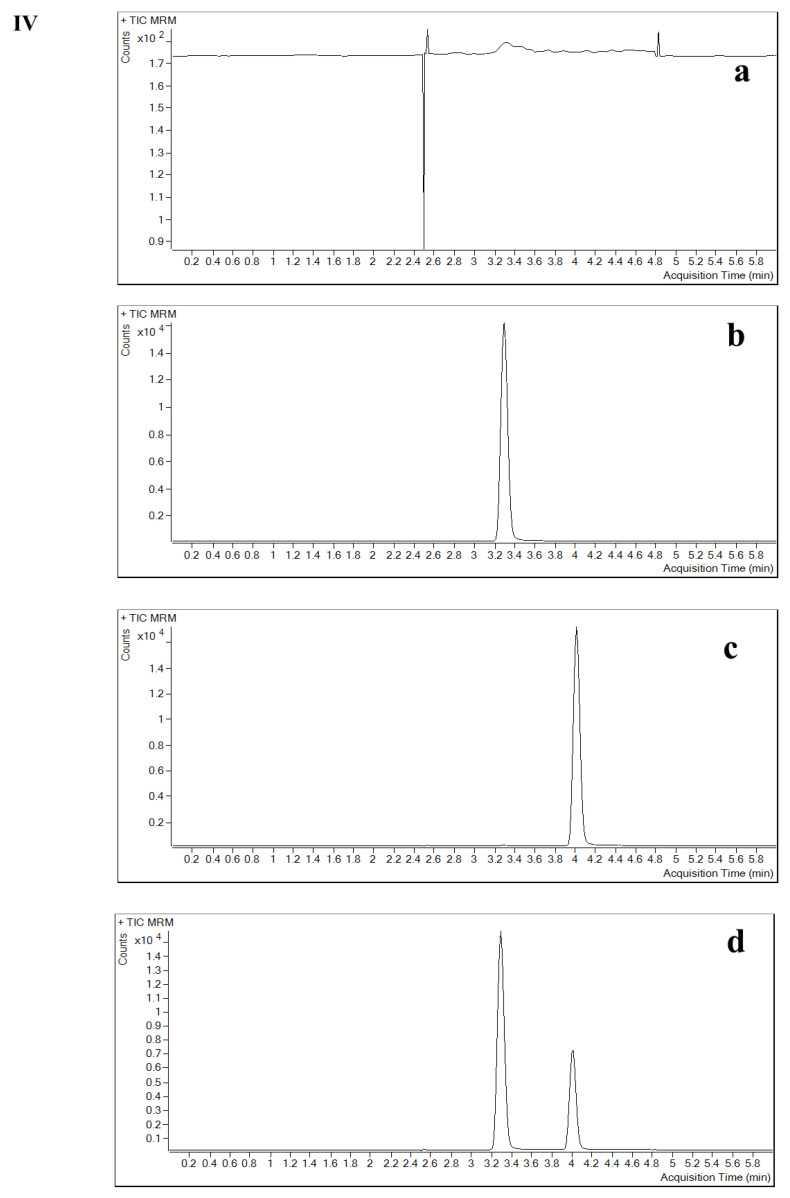

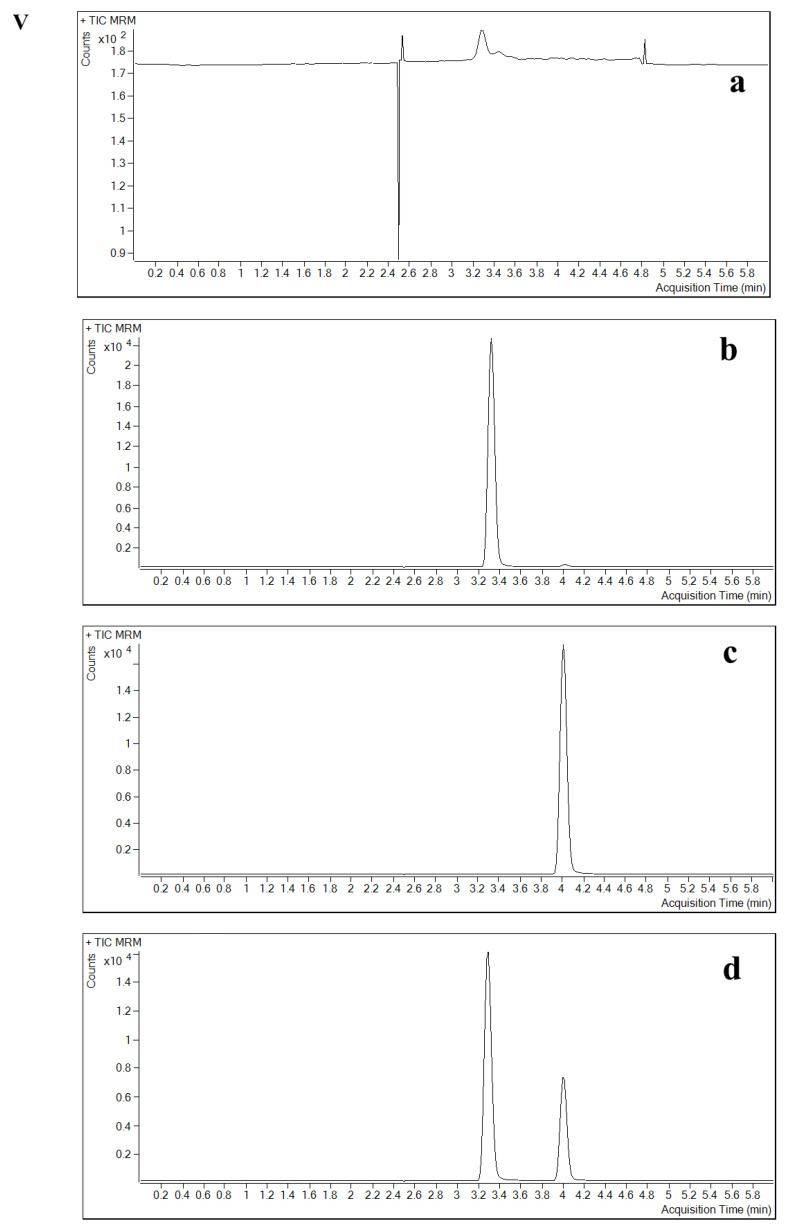

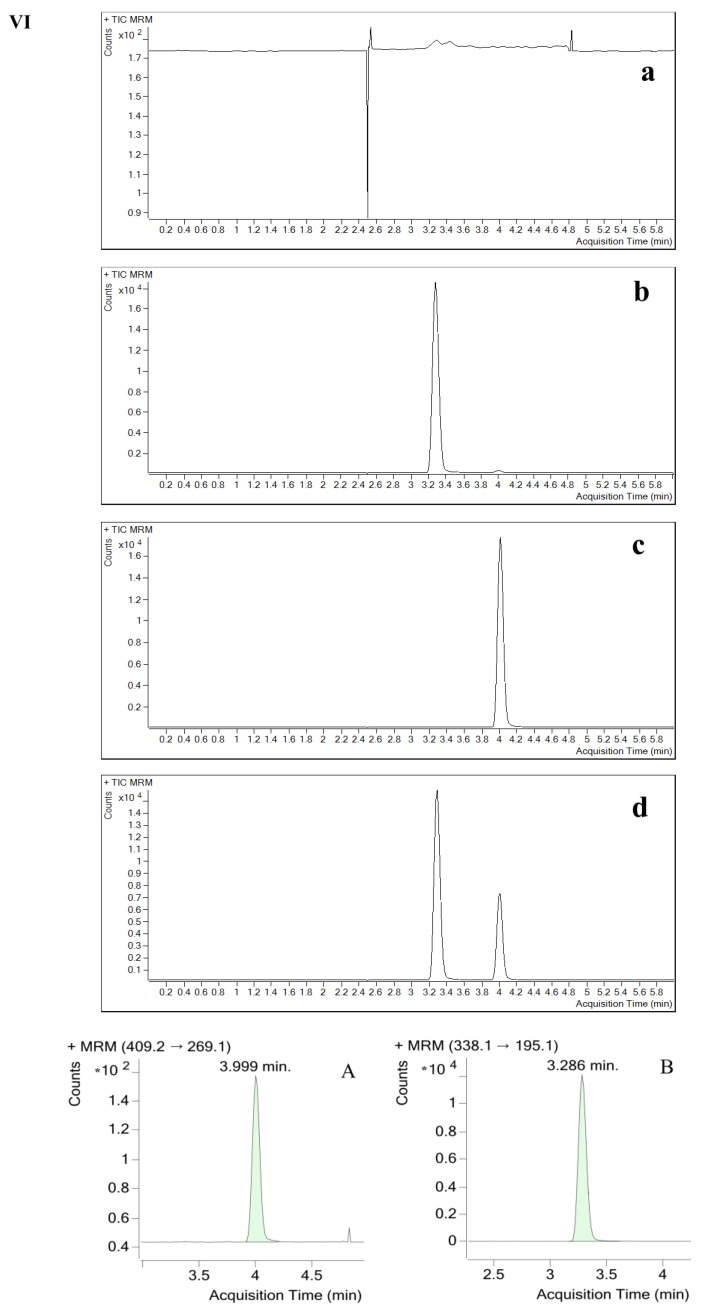
Specificity and selectivity of the method in plasma. (**a**) double blank, (**b**) single blank, (**c**) analyte only and (**d**) analyte and IS (in six different lots). Chromatograms of contezolid (**A**) and linezolid (**B**).

**Figure 3:**
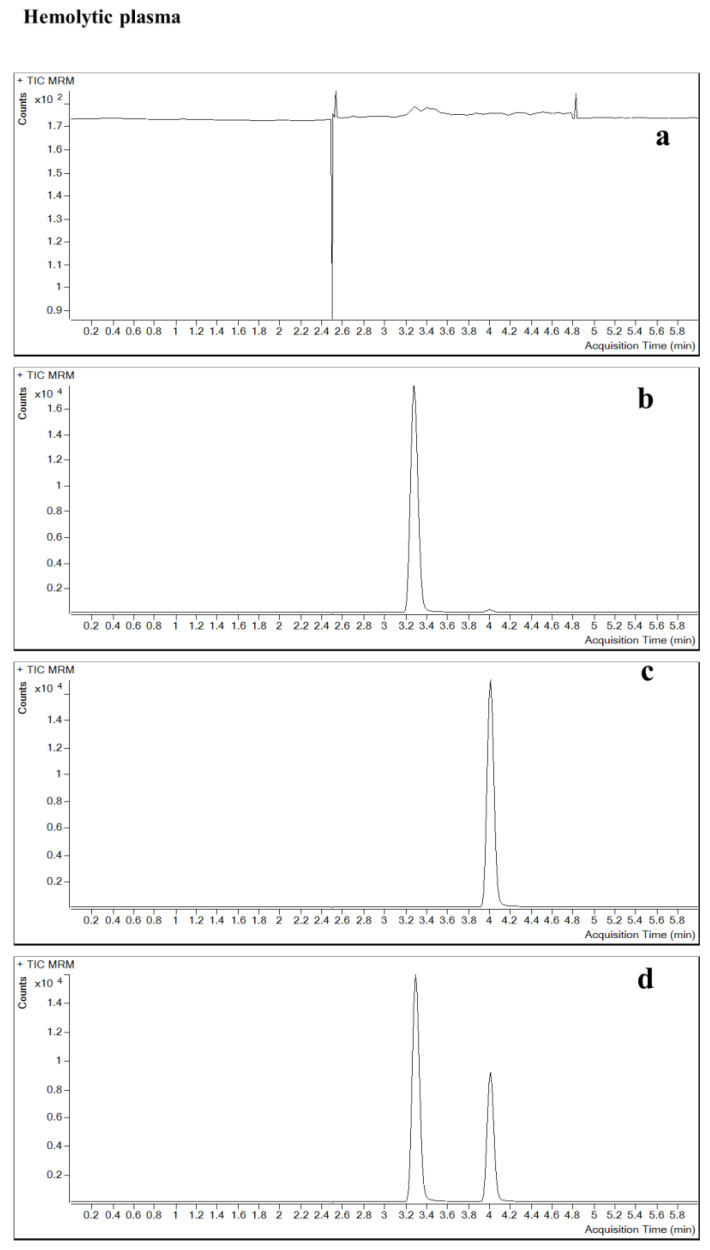

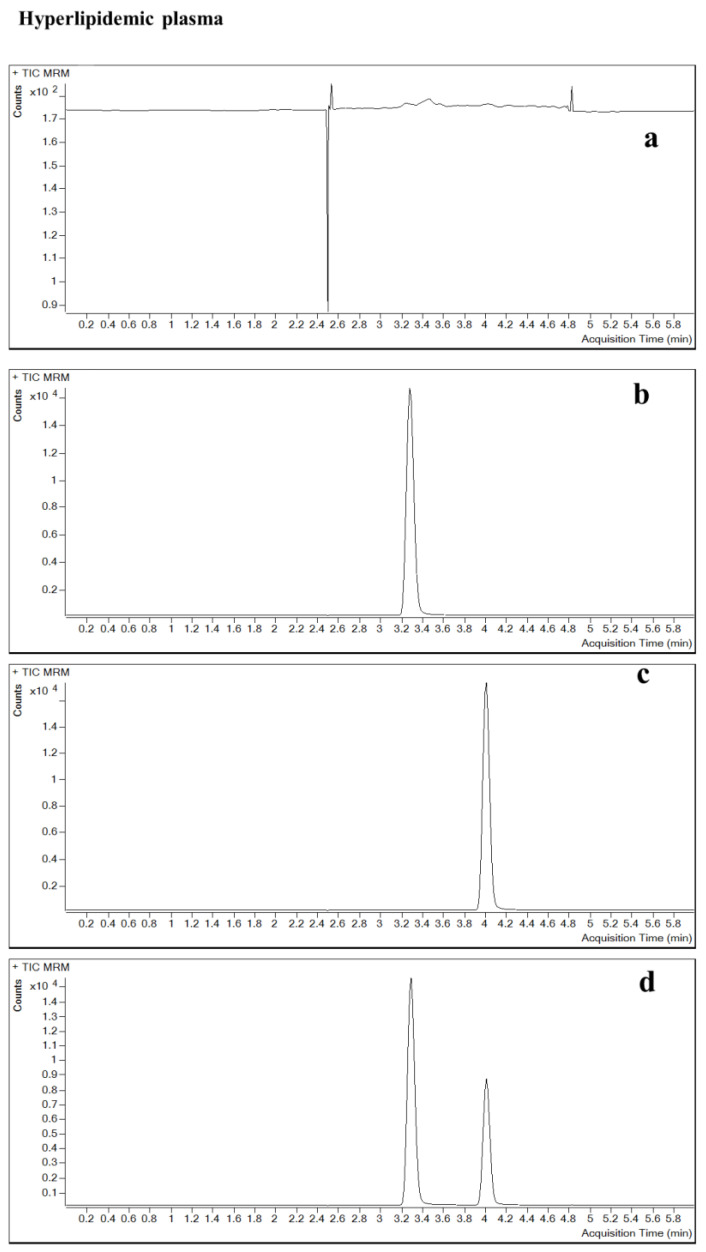
Specificity and selectivity of the method in plasma. (**a**) double blank, (**b**) single blank, (**c**) analyte only and (**d**) analyte and IS (in six different lots). Chromatograms of contezolid (**A**) and linezolid (**B**).

The specificity and selectivity results showed that the analyte or the IS in the relevant mass channels contributed less than 20% of the mean of the lower limit of quantification (LLOQ) and less than 5% of the IS response. The method produced a specific signal for the analyte, which allowed for the separation of contezolid from the IS and other components in samples. In this study, endogenous substances and IS did not affect the determination of contezolid (Figure 1, Figure 2 and Figure 3). The matrix effect (ME) of contezolid at low quality control (LQC) and high quality control (HQC) was 101.68% and 100.37% in hemolysis, as well as 104.20% and 101.00% in hyperlipidemia (Table 1).

#### 2.2.3. Sensitivity

The signal-to-noise ratio (SNR) of the LLOQs and the zero calibrators was greater than 15 in plasma and 35 in CSF. Among the LLOQs evaluated for this method, SNR reached 18.36 in plasma and 41.33 in CSF, which were considered sensitive. The mean concentration was 49.3196 (±1.3508) ng/mL in plasma and 19.9802 (±0.4460) ng/mL in CSF. The accuracy was 98.64% in plasma and 99.9% in CSF. The coefficients of variation (%CV) were 2.74% in plasma and 2.23% in CSF. The results showed that the lowest detection limits of this method were 50 ng/mL in plasma and 20 ng/mL in CSF. The results all met the criteria for acceptance.

#### 2.2.4. Precision and Accuracy

The results of precision and accuracy validation are shown in Table 2. Inter-batch precision and accuracy of the plasma samples were 0.29% to 3.37% and 93.30% to 105.59%, respectively, and intra-batch precision and accuracy were 0.10% to 1.02% and 97.34% to 103.33%, respectively. The intra-batch accuracy and precision for CSF samples were 97.57% to 107.86% and 0.63% to 5.79%, respectively, and the inter-batch accuracy and precision were 99.41% to 105.62% and 1.35% to 2.57%, respectively.

#### 2.2.5. Recovery

A simple protein precipitation method had been proved to be reliable and provided the cleanest samples. The comparison results of neat standards vs. plasma-extracted standards of contezolid and neat standards vs. CSF-extracted standards were evaluated.

The plasma recoveries of contezolid in LQC, medium quality control (MQC) and HQC were 90.14%, 95.04% and 93.63%, respectively. Their mean value was 92.94% and %CV was 2.52%. The CSF recoveries of contezolid in LQC, MQC and HQC were 102.21%, 98.05% and 93.50%, respectively. Their mean value was 97.68% and %CV was 1.47%.

#### 2.2.6. Dilution Integrity

When the concentration of the unknown sample was higher than the standard curve, a dilution study was carried out to report the accuracy and precision of the diluted sample. The samples were diluted five-fold with plasma and ten-fold with human CSF. The samples showed that the accuracy relevant error (RE) in plasma and CSF were 4.08 and −2.82.

The %CV values were 0.41 and 3.07 (Table 3). If the verified dilution multiple could not meet the requirements of testing the clinical samples, the dilution multiple would be supplemented according to the actual need.

#### 2.2.7. Stability

The stability of two concentrations of contezolid in human plasma (3.5 h at room temperature, 116 h in an automatic sampler, after three cycles from −20 °C and −80 °C to room temperature freeze-thawed, 28 days at −20 °C, and 28 and 118 days at −80 °C) and CSF (6 h at room temperature, 23.5 h in an automatic sampler, after three cycles from −20 °C and −80 °C to room temperature freeze-thawed, 90 days at −20 °C and −80 °C), had been established. The %CV values ≤ 14.22% (Table 4). We also investigated the stability of contezolid stock solution for 24 h at room temperature and 90 days at −20 °C, and the stability of IS stock solution for 24 h at room temperature and 62 days at −20 °C. The %CV values ≤ 1.96%. The results showed that the samples had good stability under different storage conditions.

#### 2.2.8. ME

As shown in Table 5, the matrix factors (MF) of contezolid at three concentrations in plasma were 97.32%, 98.33%, 98.25%, 98.00%, 94.80%, and 95.36%, respectively. Under the selected chromatographic and MS conditions, the %CVs of analytes at low, medium, and high levels were all less than 13.38%, which did not affect the quantification.

## 3. Materials and Methods

### 3.1. Reference Materials

Contezolid (99.92%) was provided by MicuRx Pharmaceuticals, Inc, Shanghai, China. Linezolid (99.7%) was purchased from ApexBio Technology LLC, Houston, TX, USA.

### 3.2. Reagents

The following reagents were purchased from Thermo Fisher Scientific (China) Co., Ltd., Shanghai China: dimethyl sulfoxide (DMSO, above 99%), ultrapure water, acetonitrile and methanol of chromatographic purity, and high-performance liquid chromatography-grade ammonium.

### 3.3. Biological Matrix

Artificial CSF (R22153) was purchased from Shanghai Yuanye Bio-technology Co., Ltd. Individual or mixed human plasma samples in K2EDTA-anticoagulated tubes were taken from healthy volunteers and stored at −10 to −30 °C. Human hemolytic and hyperlipidemia plasma samples from our hospital’s Respiratory Department were obtained and stored at −10 to −30 °C. Plasma and CSF samples were stored at −80 °C.

### 3.4. LC-MS/MS System

The chromatographic system used in the study was an Agilent 1260 high-performance liquid chromatography (HPLC) system (Agilent Technologies Inc., Santa Clara, CA, USA), consisting of a vacuum degasser, a binary pump, and an automatic sampler.

Agilent EclipsePlus C18 column (2.1 × 100 mm, 3.5 μm) was used for the elution of the analyte and IS. As mobile phases, we used 0.1% FA in a 10 mM aqueous ammonium acetate solution (A) and 0.1% FA in a 5 mM ammonium acetate acetonitrile: water (90:10, *v*/*v*) solution (B). The elution gradient started at 15% B, increased to 50% B from 0.5 min to 4.0 min, remained at 50% B until 5.0 min, and then returned to 15% B at 5.1 min. During each injection, 0.4 mL/min of flow rate was achieved, 5 μL of fluid was injected, and 6 min were spent in total.

Using an Agilent 6460A triple-quadrupole mass spectrometer (Agilent Technologies, Palo Alto, USA) connected to an electrospray ion source, positive MRM was carried out with this mass spectrometer. The nebulizer pressure was set to 30 psi. The flow of the drying gas was 11 L/min, and the drying gas temperature was held at 350 °C. The electrospray capillary voltage was optimized to 4000 V for positive and 3500 V for negative. Q1 and Q3 were both set at unit resolution. The *m/z* transition of contezolid was 409.15→269.14 (fragmentor, *F* = 80 V, collision energy, CE = 30 eV), and that of linezolid was 338.14→195.1 (*F* = 65 V, CE = 30 eV). We used the Agilent MassHunter Workstation to complete data acquisition and analysis.

### 3.5. Preparation of Calibration Standard Curves, Quality Control (QC) and IS Samples

Contezolid reference standards were precisely weighed and diluted completely with DMSO to prepare the stock solutions of 1.00 mg/mL as standard curve stock solution and the QC stock solution. The stock solutions were stored in a refrigerator of between −10 and −30 °C for future use.

Using a 50% acetonitrile aqueous solution as dilution solvent, the working standard solutions for plasma or CSF samples were prepared. The working solutions were diluted 20-fold into plasma or CSF. The concentrations of 5000, 4000, 2500, 1000, 500, 250, 100 and 50.0 ng/mL were for plasma, and 1000, 800, 600, 400, 200, 100, 40.0 and 20.0 ng/mL were for CSF calibration standards. The concentrations of 3750 (HQC), 2000 (MQC), 150 (LQC), and 50.0 (LLOQ) ng/mL were for plasma, and 750 (HQC), 350 (MQC), 60.0 (LQC), and 20.0 (LLOQ) for CSF QCs. The IS working solution, with a concentration of 800 ng/mL for plasma and 150 ng/mL for CSF samples, was prepared by diluting the IS stock solution with acetonitrile: methanol (1:1, *v*/*v*) and stored in the refrigerator at −10 to −30 °C.

### 3.6. Sample Preparation

The plasma and CSF samples (50 μL) were mixed with 250 μL of the IS working solution in 1.5 mL EP tubes. The samples were mixed for 1 min by using a vortexer, then centrifuged 10 min at 14,000 rpm (2 to 8 °C). A 100 μL plasma supernatant was added to a 200 μL acetonitrile: water (1:1) solution. After mixing, 200 μL was transferred to the HPLC vial, and 5 μL was injected into the LC-MS/MS system for analysis.

A 200 μL CSF supernatant was directly transferred to the HPLC vial, and 5 μL of the supernatant was entered into the LC-MS/MS system for analysis.

### 3.7. Method Validation and Acceptance Criteria

The method was verified according to *Bioanalytical Method Validation (M10)* of the International Council for Harmonisation of Technical Requirements for Pharmaceuticals for Human Use. The method parameters included linearity of calibration curves, selectivity and specificity, sensitivity, accuracy, precision, recovery, stability, dilution integrity, and matrix effect.

#### 3.7.1. Specificity and Selectivity

The potential interference of analyte and IS in the liquid chromatography peak region was studied by analyzing at least six normal individual blank plasma, hemolytic plasma, hyperlipidemic plasma, and six normal individual blank CSF samples, then the specificity of this method was evaluated. By analyzing a double blank (without IS or analytes) plasma and CSF, a single blank (only IS) plasma and CSF, plasma, and CSF with only analytes, as well as plasma and CSF containing analytes and IS, the specificity and selectivity of the method were determined.

The response of the interference component should not be higher than 20% of the LLOQ and not more than 5% of the IS response in each matrix LLOQ sample.

#### 3.7.2. ME

The matrix effect of three concentrations (150 ng/mL, 2000 ng/mL and 3750 ng/mL in plasma; 60.0 ng/mL, 350 ng/mL and 750 ng/mL in CSF) of contezolid in six kinds of substrates was determined. The ME is calculated from the IS normalized MF. In the sample group prepared under “sample preparation”, the peak area to mean value ratio of contezolid was recorded as A1, and the peak area to mean value ratio of IS was recorded as A2. MF = A2/A1.

The precision (the percentage of the coefficient of variation, %CV) of all individual source analytes should not be greater than 15%.

#### 3.7.3. Calibration Curve and Range

The calibration curves were prepared by using double blank samples, single blank samples (only IS), and calibration standard samples with eight concentration levels (including LLOQ and the upper limit of quantification, ULOQ).

At the LLOQ, the accuracy of back-calculated concentrations of calibrated standards should be within ±20% of the nominal concentration, while at all other levels, should be within ±15%. It is recommended that all standard curves contain at least six effective concentrations and that at least 75% of the calibration samples meet these criteria.

#### 3.7.4. Sensitivity

The LLOQ refers to the lowest level of concentration of analyte whose response is at least five-fold greater than the blank (SNR ≥ 5), whose precision is less than 20%, and whose accuracy is in the range of 80.0—120% of the nominal value.

#### 3.7.5. Precision and Accuracy

Samples of LLOQ and QC were prepared six times for three consecutive runs on three different dates and analyzed three times in each run.

Except for LLOQ, the overall accuracy of each concentration level QC sample should be within ±15% of the nominal concentration, and the accuracy of LLOQ should be less than ±20%. Except for LLOQ, the precision of each concentration level QC sample should not exceed 15% and of LLOQ should not exceed 20%.

#### 3.7.6. Carry-Over

Immediately after the high concentration of the sample (5000 ng/mL in plasma, 1000 ng/mL in CSF), the blank samples were analyzed to estimate the carry-over.

Blank samples after ULOQ should not carry over more than 20% of the LLOQ sample’s response and 5% of the IS sample’s response.

#### 3.7.7. Dilution Integrity

Contezolid (20,000 ng/mL) was added to plasma and 1750 ng/mL to CSF. These samples were diluted to five-fold of plasma or ten-fold of CSF and determined repeatedly for six times. The mean accuracy of dilution QC should be within ±15% of the nominal concentration, and the %CV should not exceed 15%.

#### 3.7.8. Stability

The stability of the stock solution, the stability of samples after frozen-thawed or treated, short- and long-term stability of samples were investigated. By analyzing the repetition of plasma samples (150 ng/mL and 3750 ng/mL) and CSF samples (60.0 ng/mL and 750 ng/mL) added under different conditions, the stability of contezolid in human plasma and CSF was evaluated. We evaluated the stability of the samples stored at room temperature (bench top stability) for six hours and compared it to the stability of samples stored in the injector for short-terms and repeated freeze-thaw cycles. A comparison was made with the concentration obtained after a period of at least 28 days in order to determine long-term stability. Through proper dilution, the stability of the stock solution was investigated by the response of the detector.

## 4. Conclusions

A simple, rapid, and sensitive LC-MS/MS method of the analysis of contezolid in human plasma and CSF without time-consuming and expensive extraction procedure was developed and validated. We also verified that it was feasible to use artificial CSF as the matrix of standard curve, which made up for the defect that it was not easy to obtain CSF from patients. By developing the method, the concentration of drugs in the plasma and CSF can be monitored and clinical treatment can be guided.

## Figures and Tables

**Table 1:** ME of the method for determining contezolid in hemolytic and hyperlipidemic plasma.

Concentration (ng/mL)	Number	Hemolytic Plasma	Hyperlipidemic Plasma
150	1	153.3127	155.2132
	2	150.9690	156.3032
	3	153.2864	157.3617
	Mean	152.5227	156.2927
	SD	±1.3456	±1.0743
	%CV	0.8800	0.6900
	Accuracy	101.6800	104.2000
	RE (%)	1.6800	4.2000
3750	1	3767.9127	3792.1579
	2	3752.9170	3775.6060
	3	3770.4290	3794.5686
	Mean	3763.7529	3787.4442
	SD	±9.4681	±10.3228
	%CV	0.2500	0.2700
	Accuracy	100.3700	101.0000
	RE (%)	0.3700	1.0000

**Table 2:** Precision and accuracy of the method for determining contezolid.

Concentration (ng/mL)	Batch	Intra-Batch (Mean ± SD)	Intra-Batch Accuracy (%)	Intra-Batch (%CV)	Inter-Batch (Mean ± SD)	Inter-Batch Accuracy (%)	Inter-Batch (%CV)
In plasma (*n* = 6)							
50.0	1	51.2670 ± 1.5182	102.53	2.96	51.6672 ± 0.5278	103.33	1.02
	2	50.9382 ± 1.7169	101.88	3.37			
	3	52.7963 ± 0.7198	105.59	1.36			
150	1	142.0762 ± 1.2834	94.72	0.90	146.0054 ± 0.8599	97.34	0.59
	2	139.9441 ± 2.4430	93.30	1.75			
	3	155.9958 ± 0.7632	104.00	0.49			
2000	1	2008.7019 ± 10.5603	100.44	0.53	2004.4452 ± 2.1689	100.22	0.11
	2	1973.4304 ± 7.4792	98.67	0.38			
	3	2031.2032 ± 11.6640	101.56	0.57			
3750	1	3712.0060 ± 14.6794	98.99	0.40	3721.1817 ± 3.8675	99.23	0.10
	2	3651.3176 ± 10.4087	97.37	0.29			
	3	3800.2216 ± 18.1291	101.34	0.48			
In CSF (*n* = 6)							
20.0	1	20.7859 ± 0.6668	103.93	3.21	21.1237 ± 0.4009	105.62	1.90
	2	21.0129 ± 0.4486	105.06	2.13			
	3	21.5723 ± 1.2258	107.86	5.68			
60.0	1	62.7186 ± 1.6451	104.53	2.62	61.3609 ± 1.4473	102.27	2.36
	2	58.7197 ± 0.8075	97.87	1.38			
	3	62.6443 ± 3.6259	104.41	5.79			
350	1	359.8353 ± 3.2945	102.81	0.92	347.9318 ± 4.6878	99.41	1.35
	2	342.4519 ± 4.3888	97.84	1.28			
	3	341.5083 ± 11.9056	97.57	3.49			
750	1	738.0302 ± 17.8846	98.40	2.42			
	2	751.4551 ± 4.7026	100.19	0.63	747.1321 ± 19.1938	99.62	2.57
	3	751.9110 ± 42.5167	100.25	5.65			

**Table 3:** Dilution integrity of the method for determining contezolid.

Number	Nominal Concentration (ng/mL)	Actual Concentration (ng/mL)	Accuracy (%)	RE (%)
In plasma (*n* = 6, diluted five-fold)				
1	20,000	20,858.37	104.29	4.29
2		20,820.85	104.10	4.10
3		20,873.87	104.37	4.37
4		20,757.35	103.79	3.79
5		20,675.17	103.38	3.38
6		20,909.58	104.55	4.55
Mean	-	20,815.87	104.08	-
SD	-	±86.25	-	-
%CV	-	0.41	-	-
RE (%)	-	4.08	-	-
In CSF (*n* = 6, diluted five-fold)				
1	1750	1739.23	99.38	−0.62
2		1747.11	99.84	−0.16
3		1757.69	100.44	0.44
4		1654.12	94.52	−5.48
5		1657.21	94.70	−5.30
6		1648.88	94.22	−5.78
Mean	-	1700.71	97.18	-
SD	-	±52.22	-	-
%CV	-	3.07	-	-
RE (%)	-	−2.82	-	-

**Table 4:** Sample stability investigation.

Nominal Concentration (ng/mL)		150				3750			
		Mean	SD	%CV	RE (%)	Mean	SD	%CV	RE (%)
In plasma (*n* = 3)									
0 h	Determined concentration (ng/mL)	154.68	±1.51	0.98		3827.04	±13.58	0.35	
	Accuracy (%)	103.12			3.12	102.05			2.05
3.5 h—at room temperature	Determined concentration (ng/mL)	154.03	±2.92	1.90		3812.67	±32.46	0.85	
	Accuracy (%)	102.68			2.68	101.67			1.67
116 h—in the automatic sampler *	Determined concentration (ng/mL)	161.21	±7.12	4.42		3819.02	±12.55	0.33	
	Accuracy (%)	107.47			7.47	101.84			1.84
Repeated freeze-thaw three times from −20 °C to room temperature	Determined concentration (ng/mL)	153.76	±1.53	1.00		3791.53	±21.06	0.56	
	Accuracy (%)	102.50			2.50	101.11			1.11
Repeated freeze-thaw three times from −80 °C to room temperature	Determined concentration (ng/mL)	152.80	±1.61	1.05		3793.19	±9.28	0.24	
	Accuracy (%)	101.86			1.86	101.15			1.15
28 days—at −20 °C	Determined concentration (ng/mL)	138.46	±2.15	1.55		3516.93	±38.64	1.10	
	Accuracy (%)	92.30			−7.70	93.78			−6.22
28 days—at −80 °C	Determined concentration (ng/mL)	135.19	±1.57	1.16		3524.58	±53.28	1.51	
	Accuracy (%)	90.13			−9.87	93.99			−6.01
118 days—at −80 °C	Determined concentration (ng/mL)	152.07	±5.64	3.71		3219.72	±46.30	1.44	
	Accuracy (%)	101.38			1.38	85.86			−14.14
In CSF (*n* = 3)									
0 h	Determined concentration (ng/mL)	62.77	±4.33	6.90		792.67	±49.44	6.24	
	Accuracy (%)	104.61			4.61	105.69			5.69
6 h–at room temperature	Determined concentration (ng/mL)	62.57	±0.64	1.03		768.33	±2.52	0.33	
	Accuracy (%)	104.28			4.28	102.44			2.44
23.5 h–in the automatic sampler *	Determined concentration (ng/mL)	60.45	±0.71	1.17		707.28	±5.09	0.72	
	Accuracy (%)	100.75			0.75	94.30			−5.70
Repeated freeze-thaw three times from −20 °C to room temperature	Determined concentration (ng/mL)	62.53	±1.07	1.71		766.00	±14.42	1.88	
	Accuracy (%)	104.22			4.22	102.13			2.13
Repeated freeze-thaw three times from −80 °C to room temperature	Determined concentration (ng/mL)	61.43	±1.59	2.59		781.33	±22.68	2.90	
	Accuracy (%)	102.39			2.39	104.18			4.18
90 days—at −20 °C	Determined concentration (ng/mL)	68.53	±0.62	0.62		734.52	±17.95	2.44	
	Accuracy (%)	114.22			14.22	97.94			−2.06
90 days—at −80 °C	Determined concentration (ng/mL)	65.62	±0.89	1.36		753.42	±6.14	0.82	
	Accuracy (%)	109.37			9.37	100.46			0.46

* The stability of the samples after treated.

**Table 5:** ME and matrix factor of the method for determining contezolid and linezolid in six individual matrices.

Number	LQC (150 ng/mL)			MQC (2000 ng/mL)			HQC (3750 ng/mL)		
	Area Ratio of Analyte	Area Ratio of IS	MF	Area Ratio of Analyte	Area Ratio of IS	MF	Area Ratio of Analyte	Area Ratio of IS	MF
In plasma									
1	0.8719	0.8896	0.9801	0.9442	0.9589	0.9847	0.9581	0.9760	0.9817
2	0.8314	0.8589	0.9680	0.9390	0.9529	0.9854	0.8791	0.8903	0.9874
3	0.8229	0.8313	0.9899	0.9110	0.9240	0.9859	0.8979	0.9153	0.9810
4	0.9893	1.0272	0.9631	0.9108	0.9319	0.9774	0.9367	0.9459	0.9903
5	0.7877	0.7994	0.9854	1.2185	1.2303	0.9904	1.2196	1.2489	0.9765
6	0.7650	0.8032	0.9524	0.9052	0.9273	0.9762	0.9204	0.9408	0.9783
Mean	-	-	0.9732	-	-	0.9833	-	-	0.9825
SD	-	-	±0.0144	-	-	±0.0055	-	-	±0.0053
%CV	-	-	1.48	-	-	0.56	-	-	0.54
In CSF									
1	0.9520	1.1002	0.8653	0.9000	0.9550	0.9424	0.8648	1.0114	0.8551
2	0.9370	0.9040	1.0365	0.9190	0.9743	0.9432	0.9371	1.0000	0.9371
3	0.9700	1.0200	0.9510	0.9429	0.9058	1.0410	0.9837	1.0343	0.9511
4	0.9970	1.0156	0.9817	0.9381	0.8822	1.0634	0.9907	1.0435	0.9494
5	0.9970	0.9265	1.0761	0.9333	0.9893	0.9434	0.9510	0.8902	1.0683
6	0.9760	1.0067	0.9695	0.9190	0.9850	0.9330	0.9604	1.0000	0.9604
Mean	-	-	0.9800	-	-	0.9777	-	-	0.9536
SD	-	-	±0.0729	-	-	±0.0582	-	-	±0.0681
%CV	-	-	7.44	-	-	5.95	-	-	7.14

## Data Availability

Data is contained within the article and supplementary material.

## Supplementary Materials

The following supporting information can be downloaded at: https://www.mdpi.com/article/10.3390/ph16010032/s1.

## Abbreviation

CE	collision energy
CNS	central nervous system
CSF	cerebrospinal fluid
CV	coefficient of variation
DMSO	dimethyl sulfoxide
ESI	electrospray ionization
FA	formic acid
HPLC	high-performance liquid chromatography
HQC	high quality control
IS	internal standard
LC-MS/MS	liquid chromatography-tandem mass spectrometry
LLOQ	lower limit of quantification
LOQ	low quality control
ME	matrix effect
MF	matrix factors
MQC	medium quality control
MRM	multiple reaction monitoring
MRSA	methicillin-resistant *Staphylococcus aureus*
PRSP	penicillin-resistant *Streptococcus pneumoniae*
QC	quality control
RE	relevant error
SNR	signal-to-noise ratio
ULOQ	upper limit of quantification
VRE	vancomycin-resistant *enterococci*
