# Supplementary material for: Development and Validation of an LC-MS/MS Method for the Quantitative Determination of Contezolid in Human Plasma and Cerebrospinal Fluid
Source: Pharmaceuticals (Basel). 2022 Dec 26;16(1):32. doi: 10.3390/ph16010032 (PMC9860615; doi:10.3390/ph16010032)
Supplement: Supplementary file 1 [file pharmaceuticals-16-00032-s001.zip › pharmaceuticals-2079097-supplementary (2).pdf]

Supplementary Materials

1. Comparison of analysis results under different conditions.

1.1 Comparison under different mobile phases.

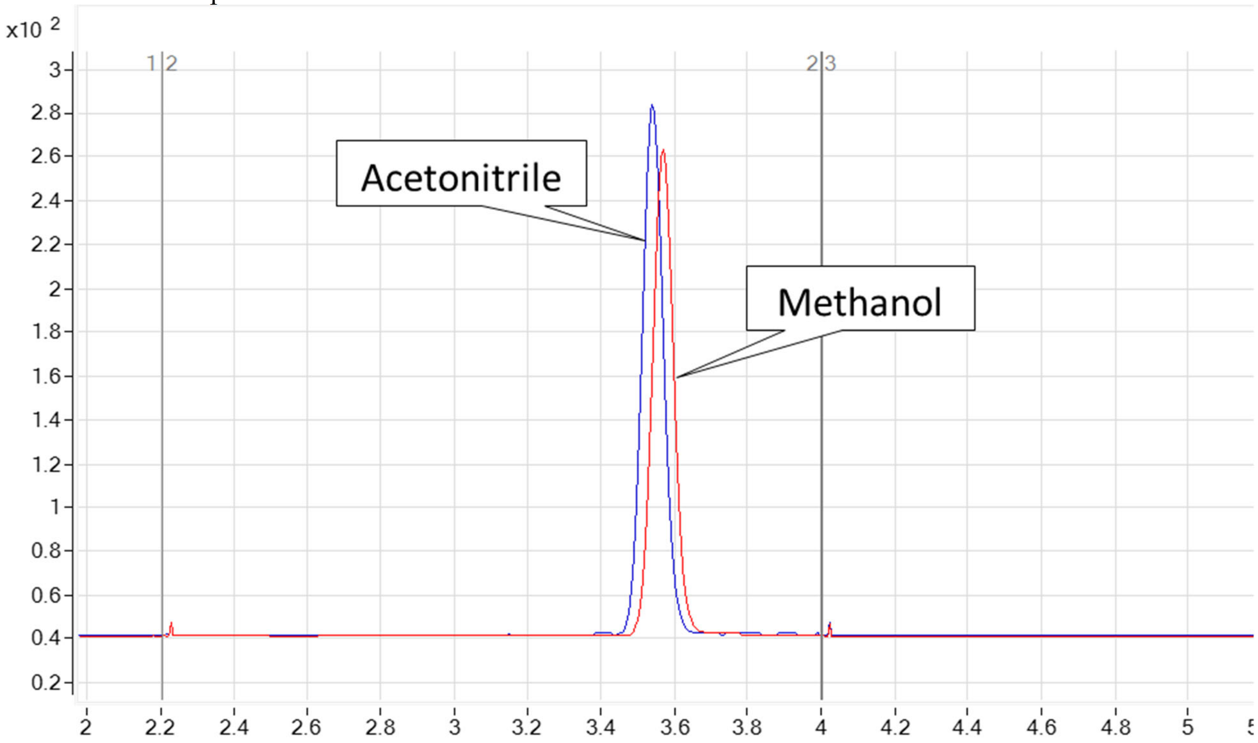

Figure S1a Comparison of chromatograms between methanol and acetonitrile as mobile phase B.

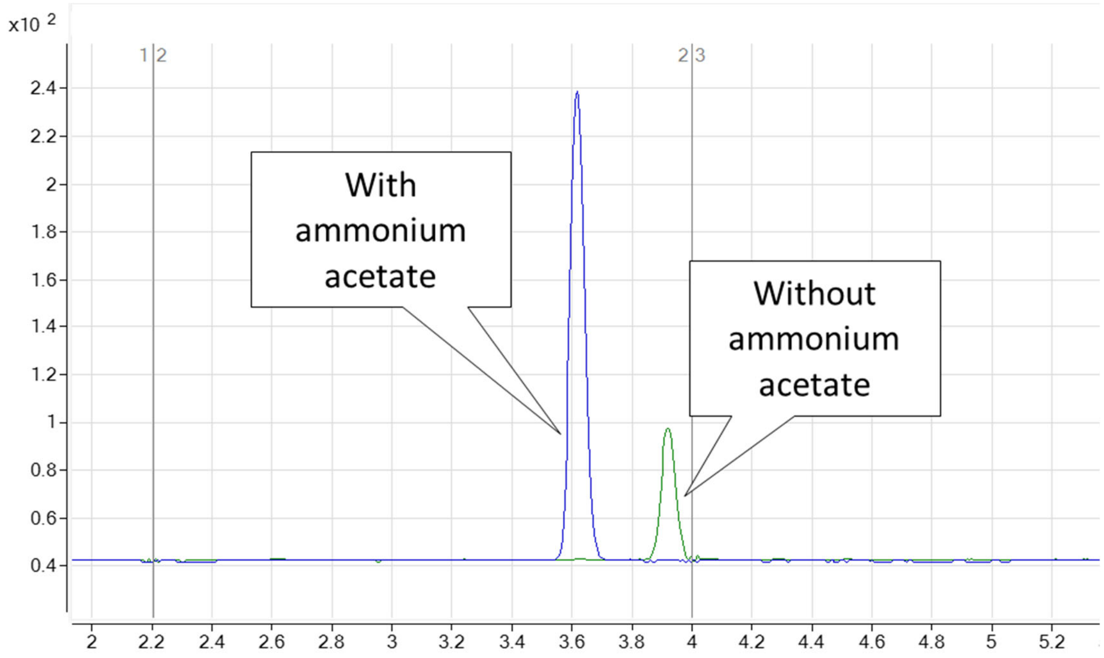

Figure S1b Comparison of chromatograms between with and without ammonium acetate in mobile phase.

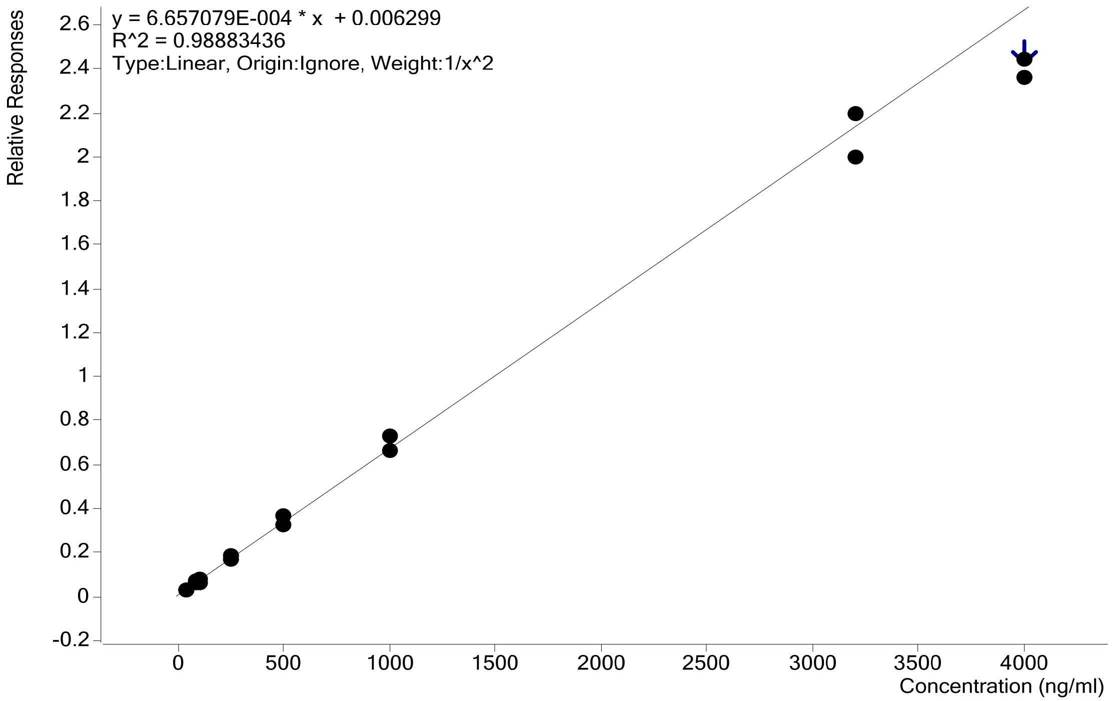

Figure S1c Calibration curve of 0.1% FA in water as mobile phase A and 0.1% FA in acetonitrile as mobile phase B.

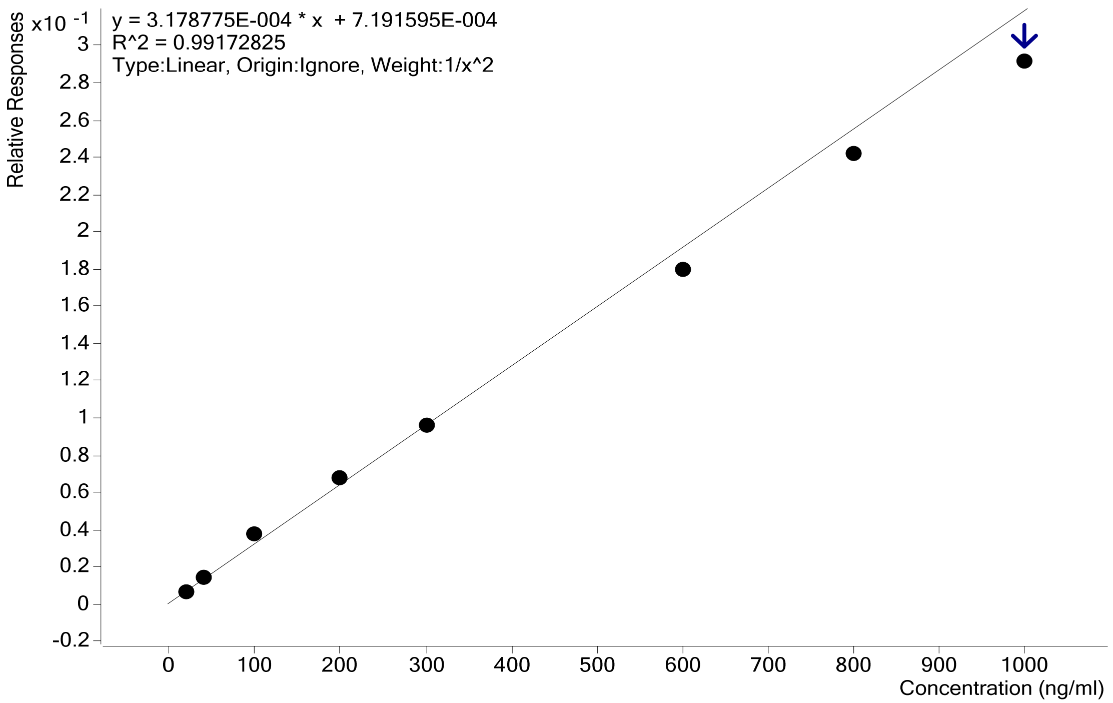

Figure S1d Calibration curve of 0.1% FA in 5 mM aqueous ammonium acetate solution as mobile phase A and 0.1% FA in 5 mM ammonium acetate acetonitrile: water (90:10, v/v) solution as mobile phase B.

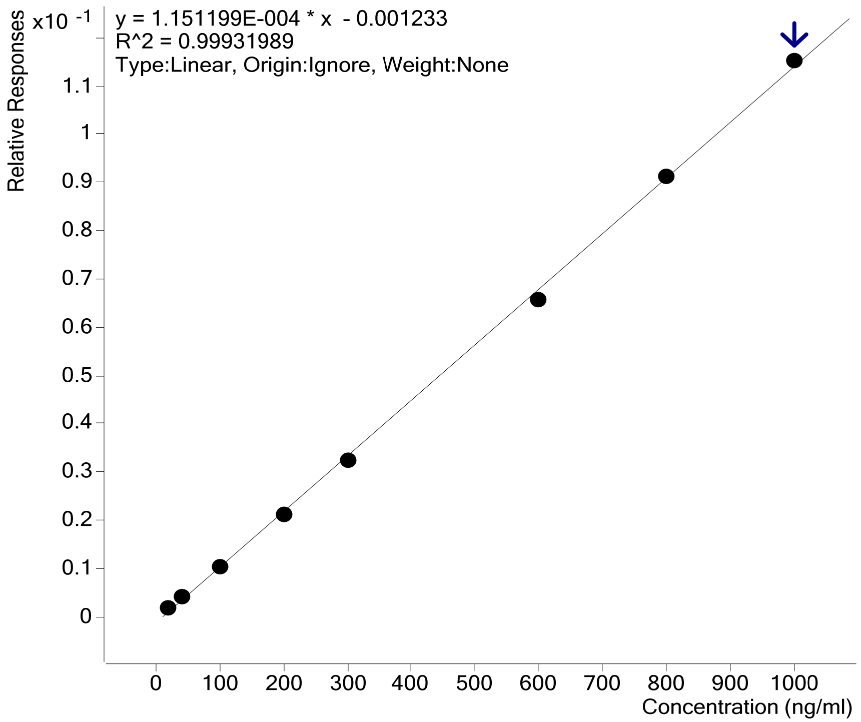

Figure S1e Calibration curve of 0.1% FA in 10 mM aqueous ammonium acetate solution as mobile phase A and 0.1% FA in 5 mM ammonium acetate acetonitrile: water (90:10, v/v) solution as mobile phase B.

1.2 Comparison between different ESI mode.

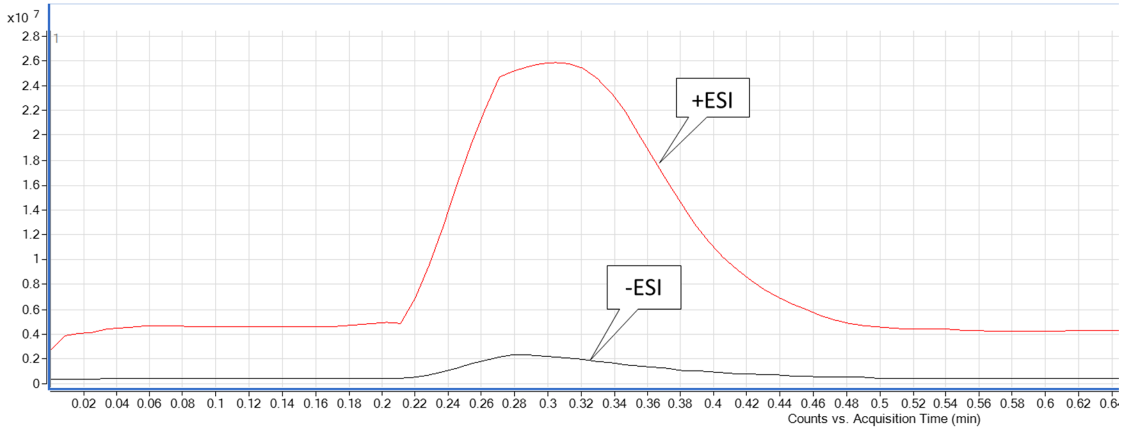

Figure S2 Comparison of chromatograms between positive ESI (+ESI) and negative ESI (-ESI) mode.

1.3 Comparison between different columns.

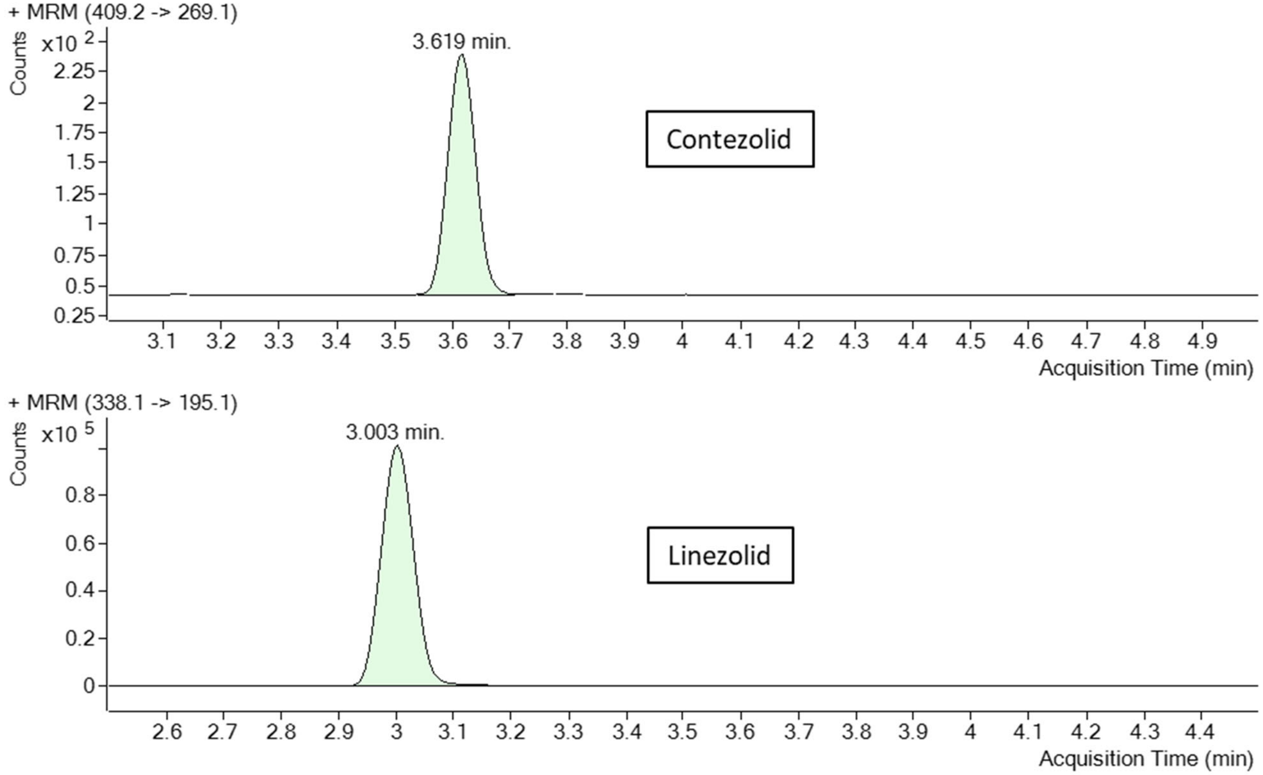

Figure S3a Chromatogram when using Eclipse Plus c18 3.5um 2.1*100mm.

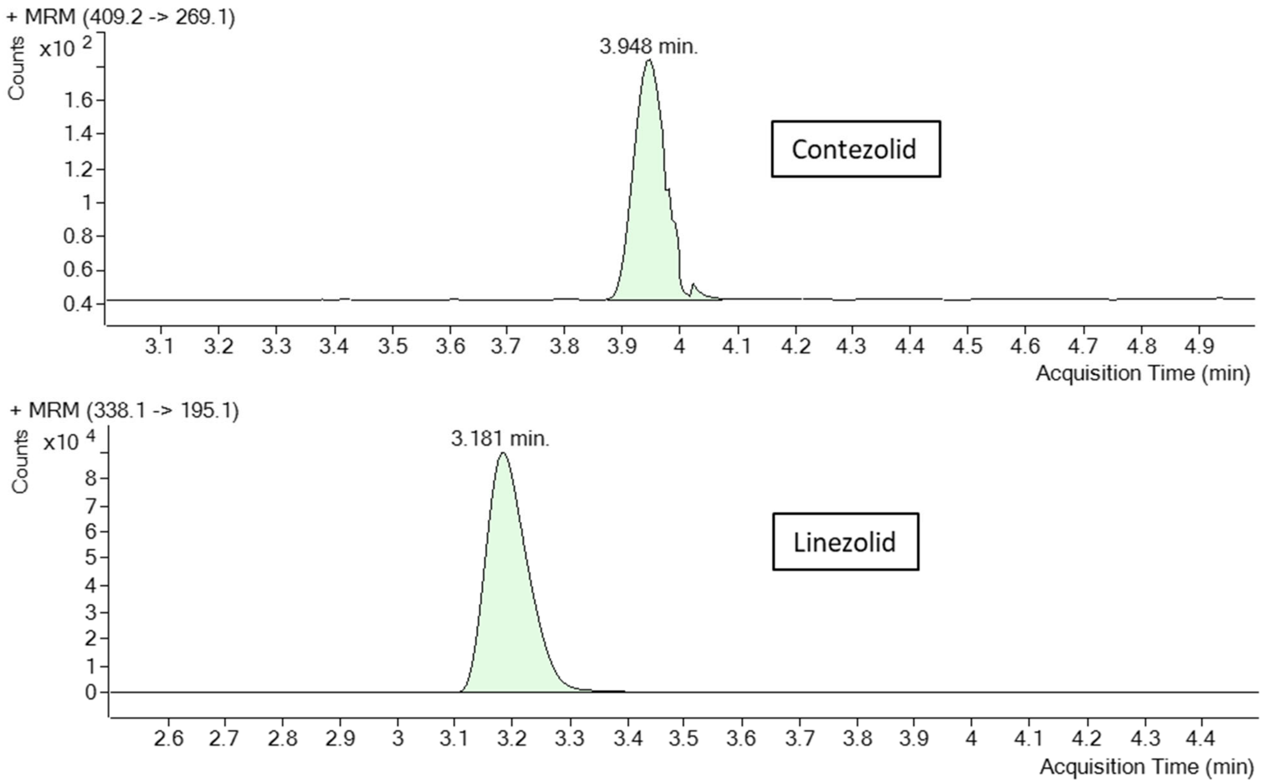

Figure S3b Chromatogram when using Zorbax SB- c18 3.5um 2.1*100mm.

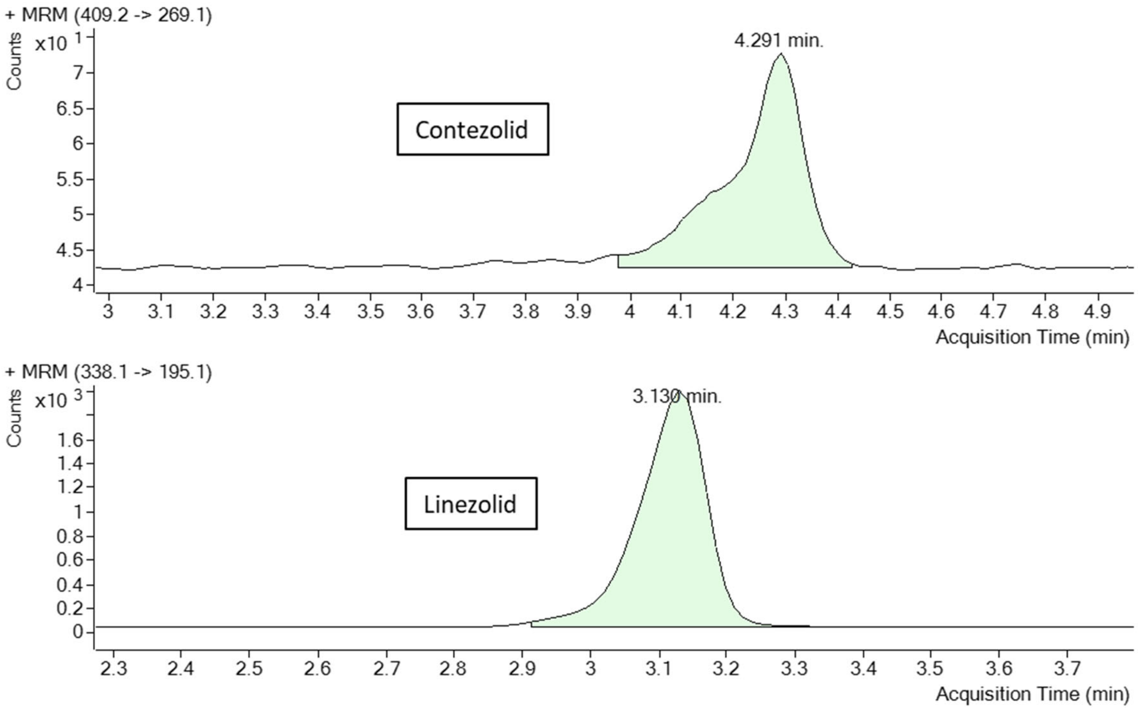

Figure S3c Chromatogram when using SB-c18 1.8um 4.6*100mm.

2. Calibration curves.

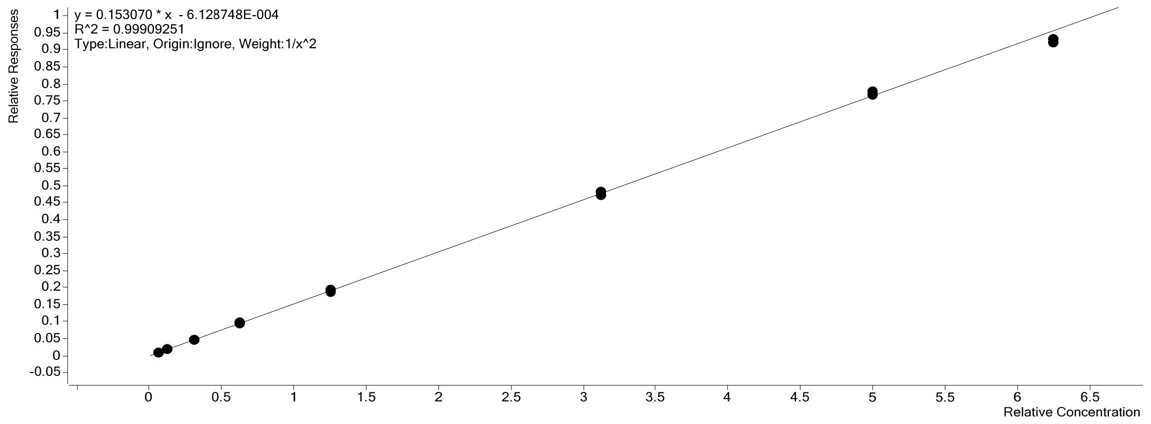

Figure S4a Calibration curve for quantitative analysis of contezolid in plasma.

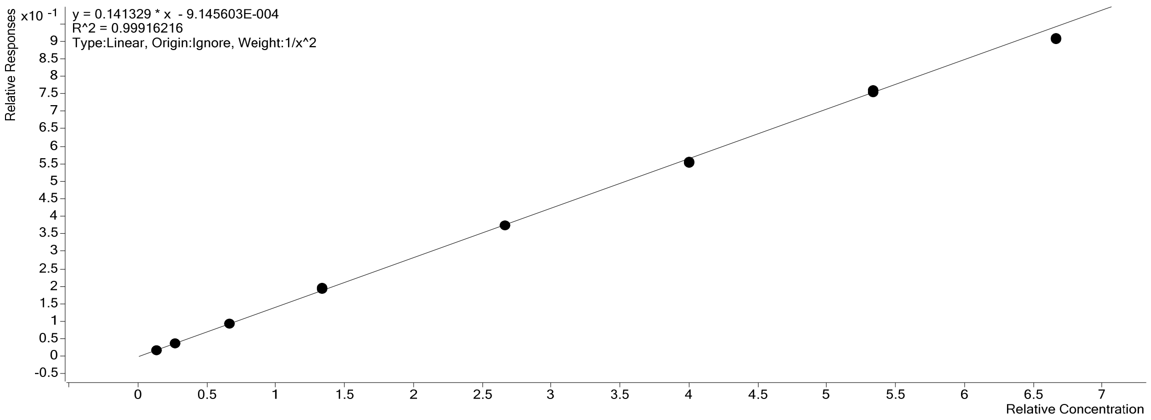

Figure S4b Calibration curve for quantitative analysis of contezolid in CSF.

3. Mass Spectrums

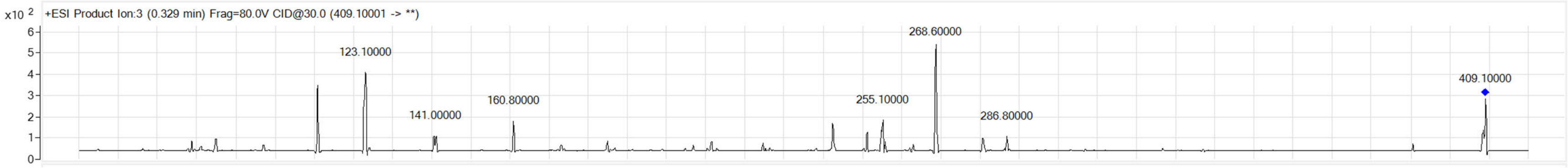

Figure S5a Mass Spectrum of contezolid.

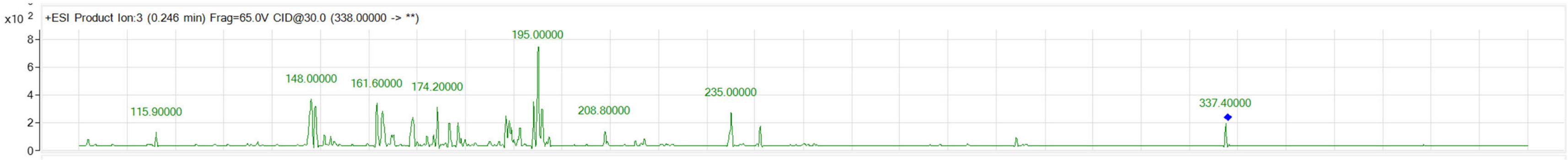

Figure S5b Mass Spectrum of linezolid.
